# DYNAMIC, BIDIRECTIONAL LONGITUDINAL RELATIONSHIP BETWEEN LEISURE ACTIVITY ENGAGEMENT AND COGNITIVE PERFORMANCE

**DOI:** 10.1093/geroni/igz038.3254

**Published:** 2019-11-08

**Authors:** Nicole Armstrong, Sarah Tom, Miguel Arce Renteria, Kaitlin Casaletto, Jennifer Weuve, Eleanor M Simonsick, Jennifer Manly, Laura B Zahodne

**Affiliations:** 1 National Institute on Aging, Baltimore, Maryland, United States; 2 Department of Neurology, Columbia University Medical Center, New York, New York, United States; 3 Taub Institute for Research on Alzheimer’s Disease and the Aging Brain, New York, New York, United States; 4 University of California, San Francisco, San Francisco, California, United States; 5 Department of Epidemiology, Boston University School of Public Health, Boston, Massachusetts, United States; 6 Longitudinal Studies Section, Translational Gerontology Branch, National Institute On Aging, NIH, Baltimore, Maryland, United States; 7 University of Michigan, Ann Arbor, Michigan, United States

## Abstract

Engagement in leisure activities, i.e., intellectual, social, and physical activities, may reduce the risk of incident dementia, yet little is known about the longitudinal, dynamic relationship between overall leisure activity engagement and cognition in older adulthood. Using data from a survey measure of 13 leisure activities, e.g., doing unpaid volunteer work and playing cards, games, or bingo, and a neuropsychological battery collected concurrently over 14 years from 2,259 multi-ethnic participants (mean age of 76.0 years) in the Washington Heights-Inwood Columbia Aging Project, we used a parallel process latent growth curve model of trajectories of both leisure activity engagement and cognitive z-scores (global cognitive performance, language, memory, and visuospatial ability). Estimates were adjusted for baseline age, years of education, sex, race/ethnicity, recruitment year, occupation (unskilled, skilled, and housewife), and baseline income. More baseline activity engagement (range, 0-13, higher indicating more engagement) was associated with higher baseline cognitive performance, i.e., global cognitive performance (estimate=0.129, standard error, SE=0.017, p<0.001), language (estimate=0.146, SE=0.020, p<0.001), memory (estimate=0.141, SE=0.025, p<0.001), and visuospatial ability (estimate=0.111, SE=0.020, p<0.001). Decline in leisure activity engagement were associated with decline in global cognitive performance (estimate=0.002, SE=0.000, p<0.001), language (estimate=0.002, SE=0.000, p<0.001), memory (estimate=0.002, SE=0.001, p<0.001), and visuospatial ability (estimate=0.001, SE=0.000, p=0.001). While both level and change in overall leisure activity engagement and cognitive performance were correlated, level of one did not predict change in the other. Similar relationships were found when examining leisure activity categories. This suggests a dynamic, bidirectional relationship between leisure activity engagement and cognitive performance among older adults.

